# Central Presynaptic Terminals Are Enriched in ATP but the Majority Lack Mitochondria

**DOI:** 10.1371/journal.pone.0125185

**Published:** 2015-04-30

**Authors:** Vrushali Chavan, Jeffery Willis, Sidney K. Walker, Helen R. Clark, Xinran Liu, Michael A. Fox, Sarika Srivastava, Konark Mukherjee

**Affiliations:** 1 Virginia Tech Carilion Research Institute, Roanoke, VA, 24016, United States of America; 2 Virginia Tech, Department of Biological Sciences, Blacksburg, VA, 24060, United States of America; 3 Yale University, School of Medicine, Department of Cell Biology, New Haven, CT, 06510, United States of America; University of Edinburgh, UNITED KINGDOM

## Abstract

Synaptic neurotransmission is known to be an energy demanding process. At the presynapse, ATP is required for loading neurotransmitters into synaptic vesicles, for priming synaptic vesicles before release, and as a substrate for various kinases and ATPases. Although it is assumed that presynaptic sites usually harbor local mitochondria, which may serve as energy powerhouse to generate ATP as well as a presynaptic calcium depot, a clear role of presynaptic mitochondria in biochemical functioning of the presynapse is not well-defined. Besides a few synaptic subtypes like the mossy fibers and the Calyx of Held, most central presynaptic sites are either *en passant* or tiny axonal terminals that have little space to accommodate a large mitochondrion. Here, we have used imaging studies to demonstrate that mitochondrial antigens poorly co-localize with the synaptic vesicle clusters and active zone marker in the cerebral cortex, hippocampus and the cerebellum. Confocal imaging analysis on neuronal cultures revealed that most neuronal mitochondria are either somatic or distributed in the proximal part of major dendrites. A large number of synapses in culture are devoid of any mitochondria. Electron micrographs from neuronal cultures further confirm our finding that the majority of presynapses may not harbor resident mitochondria. We corroborated our ultrastructural findings using serial block face scanning electron microscopy (SBFSEM) and found that more than 60% of the presynaptic terminals lacked discernible mitochondria in the wild-type mice hippocampus. Biochemical fractionation of crude synaptosomes into mitochondria and pure synaptosomes also revealed a sparse presence of mitochondrial antigen at the presynaptic boutons. Despite a low abundance of mitochondria, the synaptosomal membranes were found to be highly enriched in ATP suggesting that the presynapse may possess alternative mechanism/s for concentrating ATP for its function. The potential mechanisms including local glycolysis and the possible roles of ATP-binding synaptic proteins such as synapsins, are discussed.

## Introduction

Brain is high energy consuming in nature. Human brains account for only 2% of body weight but consume ~ 20% of total energy produced [1]. Although the brains of other animals are not nearly as energy demanding [2], a large amount of energy is still consumed in the generation of action potentials and synaptic neurotransmission [3]. Mitochondria have been shown to be present in both the pre- and post-synapse using electron microscopy. Presynaptic mitochondria were described as early as 1956 [4]. Based on early electron microscopy experiments it was postulated that there is a partial or total block of the tri-carboxylic acid (TCA) cycle in presynaptic mitochondria [5]. In fact, synaptic mitochondria have also been shown to have lower enzyme activities [6]. The morphology of presynaptic mitochondria in previously published electron microscopy (EM) studies has been reported to be varied and classified as pale and dark mitochondria [7]. At the presynapse, mitochondria are supposed to function not only as the local energy powerhouse but also as regulators for divalent ions like calcium [8] [9]. High amounts of ATP are required at the presynapse for packaging neurotransmitters into synaptic vesicles [10,11] and maintaining ionic balance as well as being a substrate for various ATPases, housekeeping proteins and kinases [12]. Neurotransmission itself is mediated by the fusion of synaptic vesicle membrane with the plasma membrane. This fusion is facilitated by a set of evolutionarily conserved molecular zippers called SNAREs [13]. The fusion event leads to the formation of cis-complexes of SNAREs, and it is believed that ATP-dependent uncoupling of these proteins is critical for their regeneration [14]. Mitochondria are also required for axonal branching [15] and for controlling the synaptic strength [16,17]. Brain subcellular fractionation studies have shown that mitochondrial proteins associate with synaptic membrane [18]. Indeed, it has been demonstrated that some of the nuclear encoded mitochondrial proteins may actually be locally translated at the presynapse [19]. Proteins responsible for trafficking mitochondria have been described [20]; also it is thought that syntaphilin is responsible for docking the axonal mitochondria [21]. Additionally, presynaptic mitochondria have been speculated to play a role in cognition and mitochondrial defects have been associated with neurodegenerative disorders [22,23]. Despite the wealth of literature on the potential function of presynaptic mitochondria in mammals, studies in mutant *Drosophila* lacking synaptic mitochondria failed to reveal specific defects in exocytosis and endocytosis at the presynapse. However, abnormalities were observed only upon intense stimulation, pertaining to mobilization of the reserve pool of synaptic vesicles [24,25]. Importantly, reconstructive 3-D electron microscopy analysis from rat hippocampus suggested that majority of presynapses (~ 59%) may lack mitochondria, raising fundamental questions about the generation of ATP at these presynaptic sites [26].

Presynaptic nerve endings are highly pleomorphic [27] and show numerous anatomical specializations including ribbon synapses, mossy fibers, and calyces of held. Most of the central synapses are however small synapses, which are either an *en passant* variety or at the termini of very small axons. Typically, the size of the central synapses is ≤ 1μm in diameter, making them difficult to study [28,29]. Therefore, most of the morphological or functional examinations have focused on specialized nerve endings which are either peripheral like the neuromuscular junction (NMJ) or central like the calyx of held and mossy fibers. The extremely small central synapses (PSD ~ 0.07 μm^2^) within the tiny bouton typically house ~ 45 recycling vesicles [30] and up to ~ 200 vesicles in total [31], whereas the medium size central synapses may accommodate more than a thousand vesicles [32]. Given that each synaptic vesicle has a diameter of ~ 40 nm, there are spatial constraints for the containment of large organelles such as mitochondria within these boutons. In this study, therefore, we investigated the fraction of small central synapses in the brain that harbor mitochondria and the total energy (ATP) level of the presynaptic compartment. Our data demonstrate that the majority of presynaptic terminals may be either devoid of mitochondrial membranes or contain relatively small mitochondria within the bouton. However, purified synaptosomal membrane preparations are highly enriched in ATP suggesting that the presynapse may possess alternative ATP concentrating and/or ATP generating mechanisms.

## Results

### Mitochondrial antigens localize poorly with synaptic vesicles and active zone marker in mouse brain

**Fig 1:**
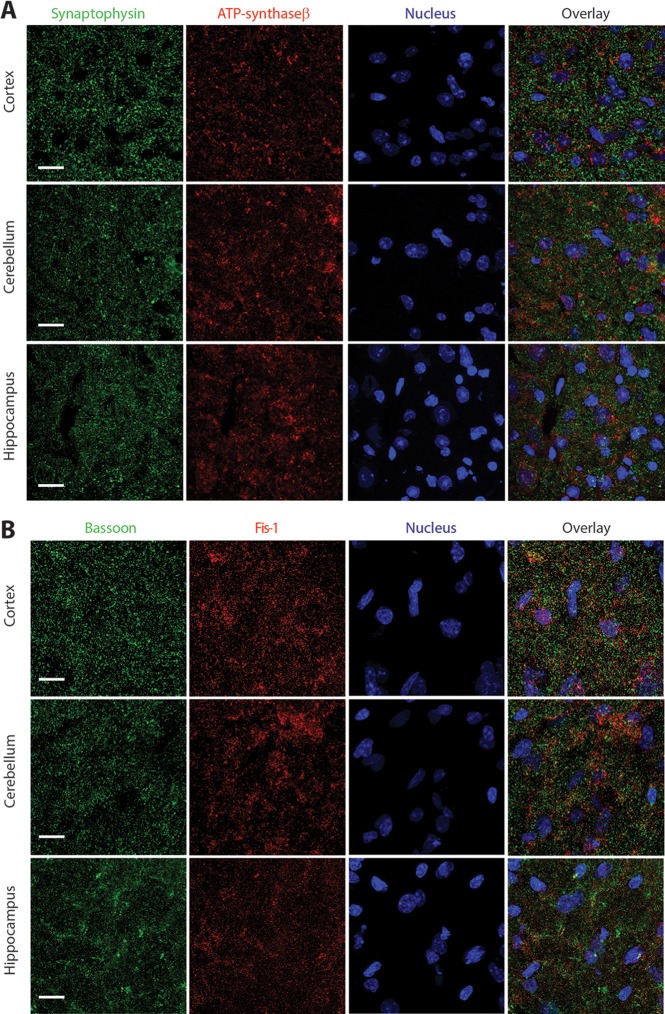
Mitochondrial markers co-localizes poorly with synaptic markers in central presynapses. A) Brain sections were stained with anti-synaptophysin (green), anti-ATP synthase β subunit (red) antibodies, and a nucleic acid Hoechst stain (blue). Images were acquired on an LSM 7 Zeiss confocal microscope. The number of red punctae overlapping with the green were quantified from 10 different sections using the Image J co-localization plugin and were estimated to be 31% ± 5% for cortex, 28% ± 3% for cerebellum and 35% ± 6% for hippocampus. Data are represented as mean ± SEM; n = 10. Representative images are from the cortex (layer 2/3), cerebellum (molecular layer) and hippocampus (stratum radiatum) as depicted; the scale bars are 15 μm. B) Brain sections were stained with anti-bassoon (green), anti-Fis1 (red) antibodies, and a nucleic acid Hoechst stain (blue). Images were acquired on an LSM 7 Zeiss confocal microscope. The number of red punctae overlapping with the green were quantified from 4 different sections using the Image J co-localization plugin and were estimated to be 29% ± 2.5% for cortex, 32% ± 6% for cerebellum and 32% ± 8% for hippocampus. Data are represented as mean ± SEM; n = 6. Representative images are from the cortex (layer 2/3), cerebellum (molecular layer) and hippocampus (stratum radiatum) as depicted. Examples of higher magnification are shown in S2 Fig.

In order to investigate the relative enrichment of mitochondria at the synapse, particularly the presynapse we focused our study on the mouse brain. Sections (20 μm thick) from adult wild-type C57BL/6 mice were stained with a marker for synaptic vesicle cluster (synaptophysin) and a mitochondrial marker (ATP synthase β subunit). Both antibodies produced a punctate pattern of staining indicating antigen clustering, as expected of organelle bound antigens (Fig 1A). The co-incidence analysis of these two markers in different regions of brain indicated that ~ 25%- 35% of synaptic vesicle clusters co-localized with the mitochondria. Specifically we looked in the layer 2/3 of cerebral cortex, stratum radiatum of hippocampi and molecular layer of the cerebellum. Results from other regions of the brain were identical. In many instances, although not co-localized, we observed an apposition of the mitochondrial antigen with the synaptic vesicle antigen (Fig 1A). This may be either due to the presence of mitochondria at the post-synapse or in the axons that are immediately proximal to the presynaptic terminal. Interestingly, the punctae sizes of both synaptophysin and ATP synthase β staining were of similar order implying that most presynapses are rather small and likely comparable in size to some extra-synaptic mitochondria. These results also indicated that the majority of synaptic vesicle clusters in the central nervous system may be spatially separated from mitochondria. The ATP synthase β-subunit has been shown to be present at the cellular or synaptic plasma-membrane [33] and multiple research groups consider myelin to be an active ATP generating site [34] [35]. Therefore, to confirm our findings we generated acute cortical slices of 300 μm thickness, in oxygenated artificial cerebrospinal fluid (ACSF). The slices were incubated with mitotracker (200 nM) for 30 min and post-fixed in 4% paraformaldehyde (PFA). These slices were then immunostained with synaptophysin antibody for subsequent imaging experiments. Confocal imaging analysis of these sections confirmed our finding that most of the central presynaptic terminals are devoid of mitochondria (S1 Fig). Since there is a possibility that some of the synaptic vesicle cluster might be non-synaptic in nature, we verified our findings by examining the co-localization of Fis1 (mitochondrial surface antigen) with bassoon, a marker for presynaptic active zone [36], [37] (Fig 1B and S2 Fig). Our data were essentially identical to those observed with synaptophysin and ATP synthase β-subunit immunostaining indicating that either the presynapses are not enriched in mitochondrial antigen or presynaptic mitochondria could not be efficiently detected by immunostaining the brain sections.

### Mitochondria are abundant in the neuronal somata and proximal compartment of major dendrites but not in presynaptic terminals

**Fig 2:**
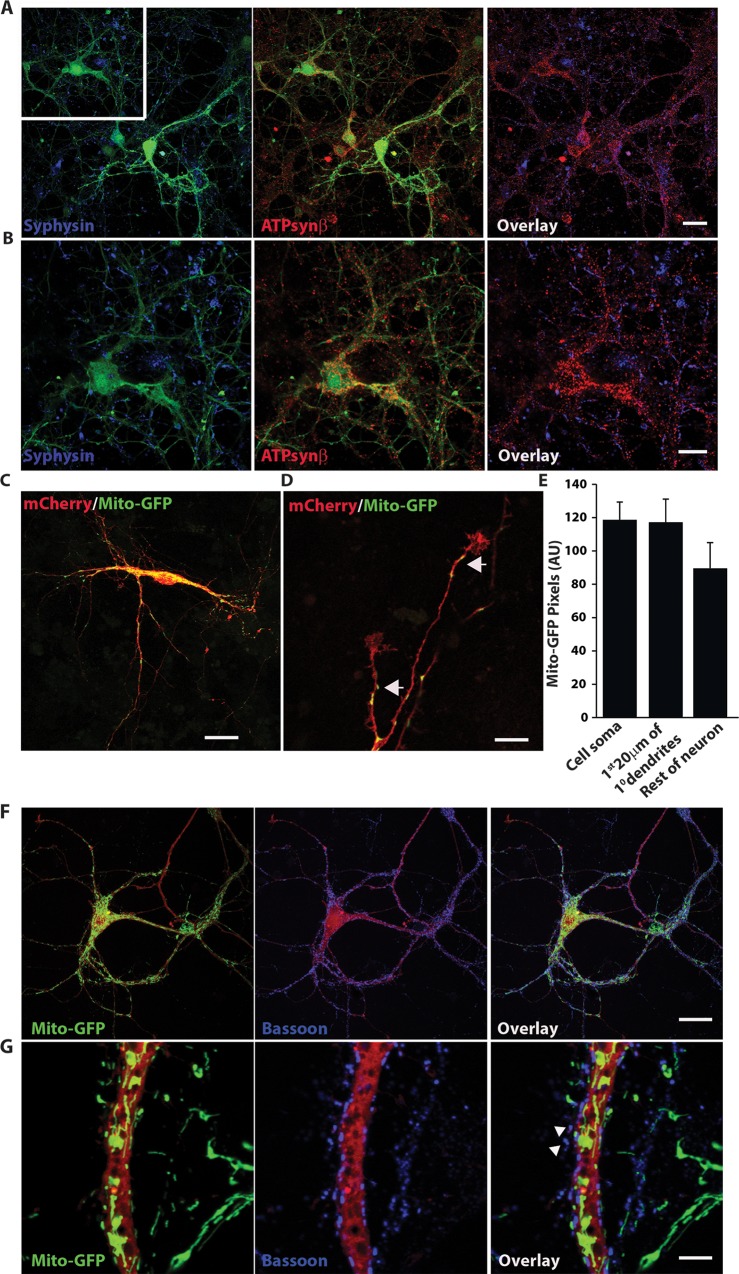
Mitochondria are located predominantly in neuronal somata and primary dendrites. A) Mixed cortical neurons were transduced with AAV-encoded GFP on the 12^th^ DIV and immunostained for synaptophysin (a pre-synaptic marker) and ATP synthase β (a mitochondrial marker) on the 14^th^ DIV. The left panel shows GFP (blue) and synaptophysin (green), the middle panel shows GFP (blue) and ATP synthase β subunit (red), and the right panel displays synaptophysin (green) and ATP synthase β subunit (red) respectively. The scale bars are 30 μm (top panel). B) The region in the white square in panel A is magnified, scale bar is 10 μm. Statistical analysis indicated that only ~ 25% ± 6% synaptophysin punctae co-localized with the ATP synthase β punctae; n = 3 cultures. C) Three dimensional construction of cultured cortical neurons co-expressing mCherry and mito-GFP plasmids. The scale bar is 40 μm. D) Representative image of collapsing growth cones that are devoid of mitochondria. The arrowheads indicate mitochondria and the white arrows point to collapsing growth cones. The scale bar is 5 μm. E) Quantification of the distribution of mitochondria in different regions of the neurons transfected with mito-GFP. Total mito-GFP pixels were quantified in the soma, the first 20 μm of primary dendrites and the rest of the neuron using Image J software. Graph displays mean value and the error bars depict SEM from n = 24. F) Low density cultures from Synapsin cre:stop tdTomato-Rosa mice. Pups were transduced with mito-GFP lentivirus at 7^th^ DIV, cultures were fixed on 14^th^ DIV and stained with a monoclonal antibody for bassoon. Confocal LSM was performed on the culture after mounting; scale bar = 40 μm. Correlation analysis indicated that ~ 23% ± 4% presynapses harbored mitochondria. G) A higher magnification image from the culture, white arrowheads indicate examples of presynapse without mitochondria. Scale bar = 10 μm. More examples of higher magnification images are also shown in S7 Fig.

In order to further verify our results at the individual neuronal level, we generated mixed cortical neuronal cultures and transduced0020030them with green fluorescent protein (GFP), using adeno-associated virus (AAV) to visualize the neurons with confocal microscopy. At 14 days *in vitro* (DIV) the cultures were fixed and immunostained with synaptophysin and ATP synthase β antibodies. Consistent with the immunostaining pattern of brain cortical sections, both antibodies produced a punctate staining pattern in cortical neuronal cultures with a low degree (~ 25%) of overlap (Fig 2A and 2B). Not surprisingly the synaptophysin punctae were mostly along the plasma membrane however, the ATP synthase β-subunit staining was particularly dense in the perikarya region suggesting that the soma is the most mitochondrial rich zone in the neurons (Fig 2A and 2B).

In order to substantiate our findings of mitochondrial distribution we decided to further investigate cultured cortical neurons using mitotracker staining. Our initial attempts to visualize the mitochondria using this staining method failed for two reasons, 1) it became very difficult to distinguish between mitochondria from individual neurons due to overlapping neurites, furthermore there were contaminating non-neuronal cells which were also stained by mitotracker and 2) we noticed cellular toxicity even at low mitotracker concentrations (i.e. 100 nM). We therefore designed a mitochondrially targeted GFP (mito-GFP) by introducing a mitochondrial leader peptide from cytochrome *c* oxidase subunit VIII at the N-terminus of GFP [38]. We first characterized our constructs by transfecting the HEK293 cells. Our results indicated that mito-GFP was exclusively localized to the membranous organelle which can be co-labeled with mitotracker red and ATP synthase β (S3 Fig), suggesting that GFP was successfully targeted to the mitochondrial compartment of the cells. We did not find any GFP staining outside of mitochondria in these cells. Next, we co-transfected the mixed cortical cultures at a very low efficiency with an mCherry encoding plasmid and mito-GFP constructs. The mCherry plasmid expresses two molecules of fluorescent protein per plasmid and is sufficiently bright to delineate the entire neuron including axons. We examined twenty four such neurons from three independent cortical cultures. Using the NIH Image J software we surveyed the distribution of mitochondria within each neuron. Our analysis indicated that more than one-third (1/3^rd^) of mitochondria are packed within the soma of the neurons (Fig 2C and 2E). Approximately forty percent of mitochondria are present in the first twenty micrometer length of the primary dendrites. The remaining mitochondria are divided throughout the distal dendrites and axons (Fig 2E). Another analysis showed a rapid decay of pixels associated with mitochondrial staining beyond 20 μm distance from the nuclei (data not shown). Taken together, our data suggests that the neuronal soma is the most mitochondrial-dense region in the neuron and may explain why there is a poor correlation between the synaptic and mitochondrial staining in brain sections. Interestingly, mitochondria in the neuronal soma are arranged in a reticular network rather than being distributed as individual entities as seen in peripheral parts of neurons (S4 Fig). These morphological differences may underlie functional differences observed between synaptic and extra-synaptic mitochondria [39]. Our transfection strategy also allowed us to take a look at the developmental relation of pre-synapse with mitochondria. Surprisingly, we found very few collapsing growth cones (~ 10%) harboring mitochondria, even though mitochondria were present in the related axons (Fig 2D). Our data suggest that in majority of cases the collapsing growth cones are also devoid of mitochondria.

In order to examine the distribution of mitochondria in respect to presynaptic active zones in a neuronal culture, we first designed a lentivirus encoding a mito-GFP. We titrated our virus to ensure near 100% gene transduction rates in both HEK293 cells and in dense neuronal culture (S5 Fig). By crossing a mouse carrying a transgenic cre-recombinase under synapsin promoter with a stop Rosa tdTomato indicator mousseline, we generated mice pups where all the cortical neurons were specifically labeled with tdTomato. We generated neuronal cultures with a low to medium density, transduced them with mito-GFP lentivirus and then examined the transduction efficiency. For our study, we only used cultures where all the neurons were transduced with mito-GFP (S6 Fig). This was essential in order to ensure that every presynaptic terminal that we observed was contributed by a neuron that was expressing mito-GFP. We stained these cultures at 14 DIV with bassoon antibody and performed confocal microscopy analysis. Consistent with our findings obtained with ATP synthase β staining, the GFP overexpressing mitochondria were found to be highly enriched in soma (Fig 2F). Moreover, we observed that a large number of presynapses (~ 76%) lacked mitochondria (Fig 2F and 2G and S7 Fig) further suggesting that most presynapses are not mitochondria enriched.

### Ultrastructural analysis revealed that the majority of presynaptic terminals lack discernible mitochondria

**Fig 3:**
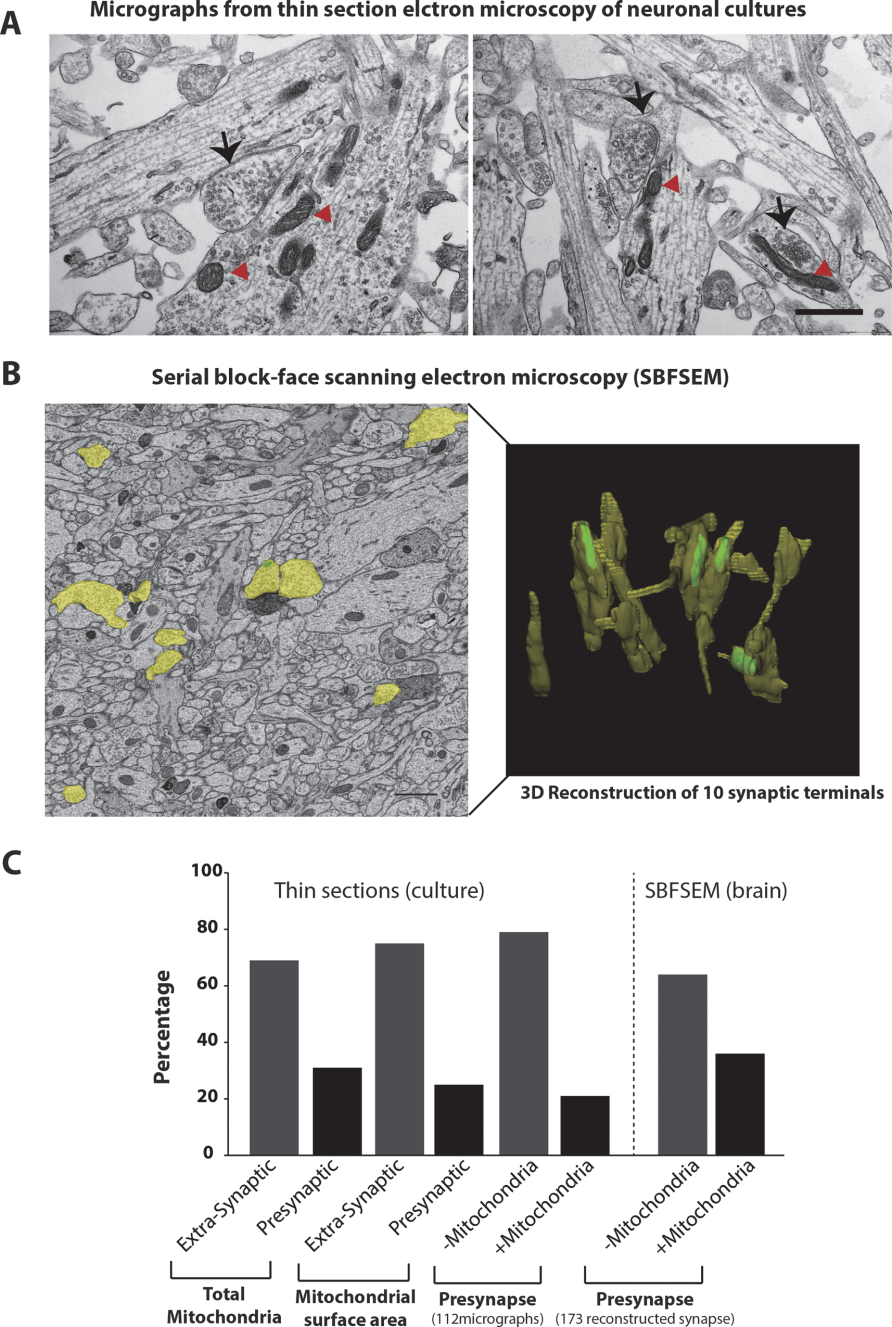
Ultrastructural analysis revealed a low abundance of mitochondria at presynaptic terminals. A) Mixed cortical cultures were fixed on the 14^th^ DIV. The cultures were prepared for electron microscopy as described in the materials and methods section. Two representative micrographs used for analyzing the distribution of mitochondria are depicted. Black arrows point to the presynaptic terminals with synaptic vesicles, red arrow head indicates the mitochondria observed at these nerve terminals. B) Serial block face scanning electron microscopy or SBFSEM analysis from hippocampi of P15 wild-type mice. Left panel depicts a representative 2D ultramicrograph from the dataset (scale bar = 1000 nm); right panel depicts 3D reconstruction of 10 presynaptic terminals. Only four out of 10 presynaptic terminals showed discernible mitochondria. C) Histograms showing quantitation from 112 micrographs obtained from thin section TEM analyzed using the Image J program and from 173 reconstructed presynaptic terminals obtained by SBFSEM and analyzed using the TRAKEM2 software.

Since our imaging analysis in neuronal cultures revealed that ~ 25% of mitochondria may be present outside of soma and proximal dendrites, and since ~ 75% of presynapses in culture seemed to lack mitochondria, we decided to use transmission electron-microscopy to further investigate the precise localization of these mitochondria. We analyzed 112 ultramicrographs from 12 different culture wells derived from two different mice. In choosing our micrographs we ensured that each had at least one presynaptic terminal, as recognized by the presynaptic vesicle cluster and postsynaptic membrane, and no neuronal soma (which is characterized by the presence of a nucleus). We quantified the electron micrographs using the following criteria 1) the percentage of extra-somatic mitochondria that reside in a presynapse, 2) the total membrane area of the observed synaptic and extra-synaptic mitochondria, and 3) the number of synapses that harbor mitochondria. Consistent with our confocal imaging data, we found that the majority (~ 65%) of the non-somatic mitochondria are also extra-synaptic (Fig 3A and 3C). Importantly, the mitochondria present in the presynaptic bouton were typically smaller in size than the extra-synaptic mitochondria which was also reflected in our analysis of the total surface area of the mitochondrial membranes (Fig 3C). Our analysis further indicated that only ~ 20% of the presynapses in the electron micrographs of neuronal culture displayed mitochondrial sections indicating that presynapse may not be particularly enriched in mitochondria (Fig 3C).

**Fig 4:**
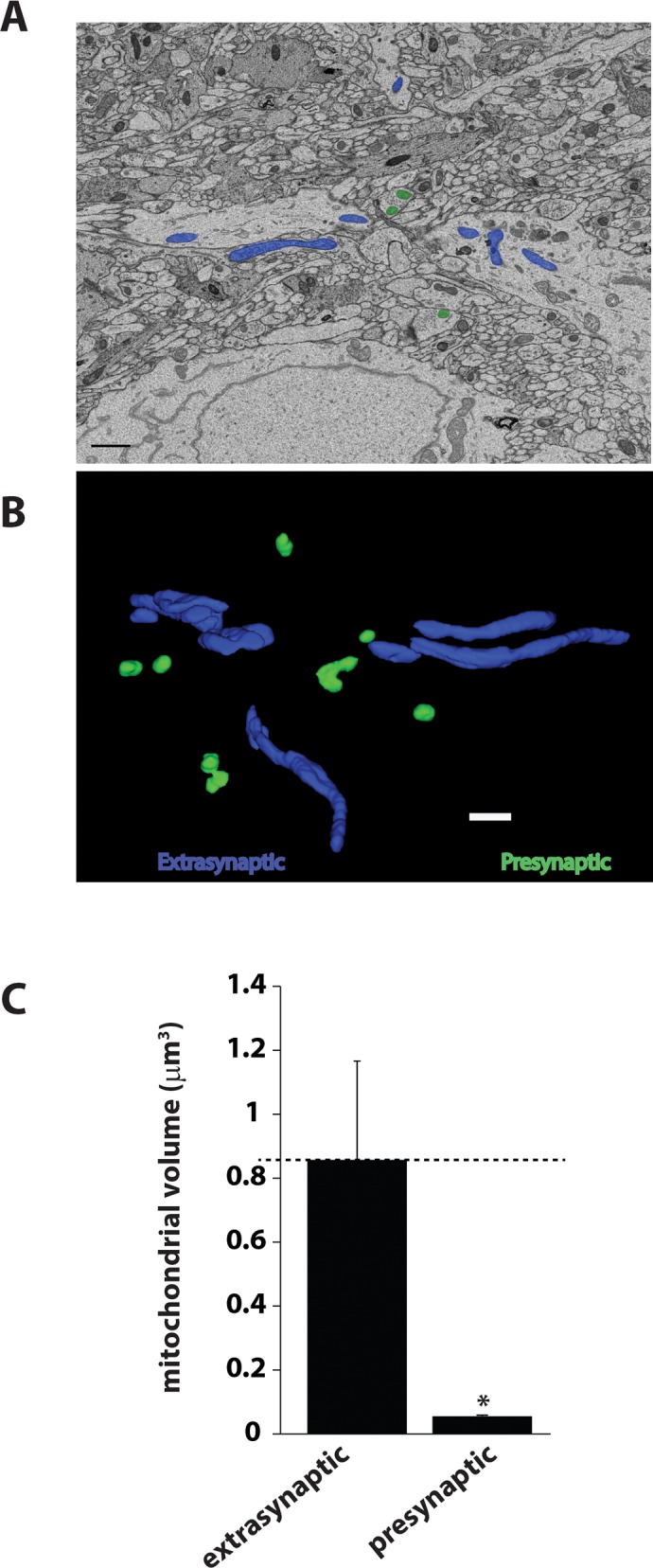
Presynaptic mitochondria are relatively small in volume compared to somatic mitochondria. A) Representative 2D image from the SBFSEM dataset showing extrasynaptic mitochondria in blue and presynaptic mitochondria in green. B) 3D reconstructions of presynaptic and extrasynaptic mitochondria using TRAKEM2 software. Scale bar = 1000 nm. C) Bar graph depicting the volume of extrasynaptic and presynaptic mitochondria. Data are plotted as mean ± SEM. n = 3 different sets.

It is possible that our ultrastructural analysis may have underestimated the number of presynapses that harbor mitochondria since they are ultra-thin sections. Importantly, synapses formed *in vitro* may be significantly different from those formed *in vivo*. We therefore next analyzed image datasets acquired by SBFSEM from wild-type mice hippocampi using the Fiji software package. We tracked and reconstructed 173 clearly visible presynaptic nerve terminals based on the presence of synaptic vesicles and postsynaptic membrane (Fig 3B). Upon analysis it was evident that only ~ 36% of presynapses actually harbored mitochondria (Fig 3B and 3C). These findings are consistent with the previous electron microscopy studies [26] and revealed a slightly higher fraction of pre-synaptic mitochondria compared to the results obtained with the co-localization studies in brain sections. The possible explanations for a slightly smaller number of presynaptic mitochondria detected by confocal imaging may be that the size of presynaptic mitochondria are much smaller compared to the extra-synaptic mitochondria (Fig 4A,4B and 4C) making it more difficult to be detected by immunofluorescence.

### Brain biochemical fractionation studies show the presynapse is extremely ATP-rich

**Fig 5:**
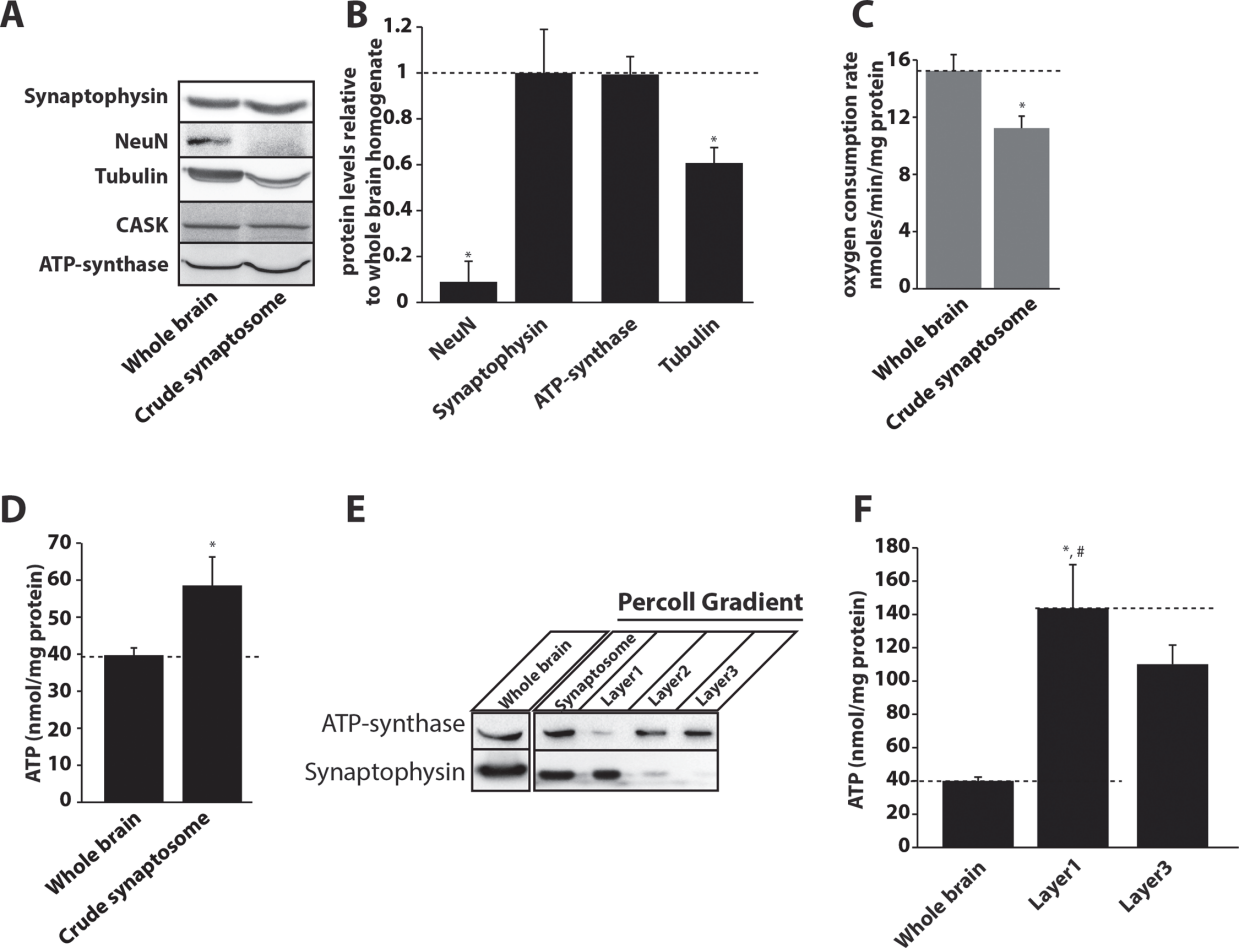
Neuronal presynapses are highly enriched in ATP. A) Immunoblots from whole brain and crude synaptosomal fractions detected with the specified antibodies. B) Relative protein quantification from three different synaptosomal preparations compared to the whole brain. (Absolute value for whole brain is depicted as 1). Data are plotted as mean ± SEM. C) Mitochondrial oxygen consumption rate in crude synaptosomes and whole brain homogenates. Total oxygen consumption was normalized to the protein levels in both fractions. D) Total ATP levels in crude synaptosomes and whole brain normalized to the protein concentrations in each fraction. E) Percoll density gradient separation of mitochondrial and synaptosomal fractions. Synaptophysin was used as a marker for synaptosomes and ATP synthase β-subunit was used as a marker for mitochondria. F) Total ATP levels detected in the whole brain homogenate, synaptosomal and mitochondrial membrane-rich fractions obtained from the Percoll density gradient method. Data were normalized to total protein levels in each fraction and plotted as mean ± SEM.

Homogenized brain is used as a source of both mitochondria and synaptosomes [40,41]. We first generated crude synaptosomes (isolated nerve terminals) from the post-nuclear supernatant fractions. We loaded equal volumes of the whole brain and crude synaptosomal proteins and performed the immunoblotting procedure using nuclear, cytoskeletal, synaptic, and mitochondrial specific markers. As can be seen in Fig 5A and 5B the nuclear and cytoskeletal antigens were reduced by ~ 80% and ~ 40% respectively in the crude synaptosomal preparation. However, no decrease in the levels of the synaptic and mitochondrial specific antigens was observed indicating a selective enrichment of these two fractions. Whole brain homogenates have been utilized by multiple groups for measuring mitochondrial respiration and were demonstrated to be a good preparation for measuring oxygen consumption by brain mitochondria [42,43]. We therefore compared the total oxygen consumption rates of whole brain homogenates and crude synaptosomes to investigate any major difference in the functioning of these two preparations. However, despite being mitochondria-rich, the crude synaptosomes exhibited a ~ 20% reduction in their oxygen consumption capacity compared to the whole brain homogenates (Fig 5C). This may be due to two reasons 1) the whole brain homogenate contains endogenous substrates which may stimulate mitochondrial respiration, and 2) the mitochondrial membranes in the crude synaptosomal preparation may have been relatively more damaged during the homogenization procedure. We next tested the total ATP levels (normalized to protein concentration) in both whole brain homogenate and synaptosomal fractions using a stabilized luciferase-based assay. Intriguingly, we found that crude synaptosomes are ATP enriched compared to the whole brain homogenate (Fig 5D). Increased levels of ATP in synaptosomes may be attributed to a higher rate of glycolysis [44] however, lactate levels in the whole brain and crude synaptosomes were found to be identical (S8 Fig).

Since mitochondria in crude synaptosomes may be either within the synaptosomal compartment or free floating, we next separated these fractions using a Percoll density gradient method as previously described [41]. Crude synaptosomes were carefully layered on a step gradient of isotonic Percoll and centrifuged as described in the materials and methods. Crude synaptosomes separated into four density gradient layers. The topmost layer was myelin-rich and was discarded. We analyzed the remaining three gradient layers, of which the first layer (layer 1) was strongly positive for the synaptic vesicle antigen synaptophysin and the third layer (layer 3) was strongly positive for the mitochondrial antigen ATP synthase β (Fig 5E). The second layer (layer 2) was positive for ATP synthase β, but also contained minor quantities of synaptophysin indicating that these mitochondria were contaminated with the synaptic membranes. In-line with our imaging and ultrastructural data, the synaptophysin positive layer had only minor quantities of ATP synthase β, which demonstrates that there were very few mitochondria present in the pinched nerve endings (Fig 5E). We next used a stabilized luciferase-based assay system to quantify the total ATP levels associated with the purified synaptosomal and mitochondrial-rich fractions. As expected, the mitochondrial fraction exhibited elevated levels of ATP as compared to the whole brain homogenate; however, the synaptosomal fraction contained even higher amounts of ATP compared to the mitochondrial fraction (Fig 5F) indicating that synaptosomes may possess an alternative mechanism of concentrating or generating ATP. Taken together our data suggest that even though a majority of presynaptic terminals lack mitochondria, they utilize alternative mechanism/s to generate and store the high concentration of ATP that is required for neurotransmission.

## Discussion

Neurotransmission is a metabolically active process which contributes immensely to the total energy consumption by nervous tissue. Our data demonstrate that although the presynapse is sparsely populated with mitochondrial membranes, it is extremely ATP rich. Our findings may also provide a possible explanation for the experimental observations that abrogating mitochondrial trafficking does not affect basal neurotransmission in dMiro and drp1 mutant flies [24,25].

How do synaptosomes harboring only a minor fraction of mitochondrial membranes contain high amounts of ATP? One possibility may be high rates of local glycolysis due to abundance of glycolytic enzymes present in the presynapse [45]. However, we did not find higher amounts of lactate (a product of anaerobic glycolysis) in the crude synaptosomal preparations (S8 Fig) indicating that either increased lactate may rapidly be catabolized in synaptosomes or increased glycolysis may not be the likely cause of high ATP levels. Alternatively, lactate generated at the presynapse may be secreted into extracellular space. It has been shown that rodent neurons express high levels of the monocarboxylate transporter 2 (MCT2) which is important for lactate influx into neurons. However, neurons are known to release as much lactate as astrocytes [46]. In fact, it has also been suggested that in dendritic spines MCT2 may participate in efflux of lactate generated by intense glycolysis [44]. It is feasible that a similar mechanism may occur at the presynapse also. It has been noted that a GAPDH inhibitor reduces depolarization-induced glutamate release, whereas oligomycin, an inhibitor of mitochondrial ATP synthase does not alter glutamate release [45]. These data indeed indicate that glycolysis may be directly responsible for higher ATP levels at the presynapse. However, recent studies do not agree with the idea of glycolysis as the primary contributor of ATP at the presynapse [47].

There are two other possible mechanisms of ATP abundance at the presynapse: 1) many clear core synaptic vesicles are themselves known to package ATP in their lumen [48] and 2) a number of synaptic vesicle proteins and active zone-associated proteins are known to bind ATP without any evidence of ATP hydrolysis. It has been shown that synaptic vesicle 2 (SV2) and the SV2- related protein homolog (SVOP) are both capable of binding ATP [49,50]. SV2 can bind to two adenine nucleotides and has been speculated to be a possible contributor in ATP-dependent priming [49]. Similarly, the highly abundant synaptic proteins synapsin I, II and III are also known to bind ATP with high affinity [51,52] although they differ in the effect of the divalent ion calcium on this interaction. The binding of ATP to synapsin I is calcium-dependent, the binding of ATP to synapsin II is calcium-independent, and the binding of ATP to synapsin III is inhibited by calcium [51]. Although these proteins bind ATP like SV2, no ATPase activity has yet been demonstrated. Mice lacking synapsins I and II exhibit a severe synaptic depression upon repetitive stimulation. This phenotype is manifested in both synapsin I and synapsin II double knockout mice as well as in synapsin II knockout mice [53]. It has also been suggested that synapsins may function in the maturation of vesicles at the active zone [53] and a lack of ATP may explain the lack of vesicle maturation in synapsin mutant mice. Besides synaptic vesicle proteins, the active zone protein liprin-α is also known to bind to ATP but not GTP [54]. However, the functional relevance of this binding has yet to be investigated.

In conclusion, the most intriguing aspect of our findings is that we observed a clear biochemical separation of the ATP-enriched synaptosomal fraction from the mitochondrial fraction in crude synaptosomal preparations, suggesting that the association of large amounts of ATP with presynaptic membranes likely requires an ATP-generating/enriching step. This enrichment could either be due to recruitment of glycolytic enzymes by the synaptic vesicles or evolutionary recruitment of ATP-binding molecules at the presynapse, a combination of these mechanisms also remain a distinct possibility to be further investigated.

## Materials and Methods

### Antibodies

Monoclonal antibodies against ATP synthase β and bassoon were procured from Mitosciences and Enzo lifesciences respectively. Polyclonal antibodies against synaptophysin and Fis1 were obtained from Sigma and Pierce respectively. Aldehyde fixable mitotracker, MitoTracker Red CMXRos was obtained from Life technologies.

### Mice

C57BL6 wild-type mice were maintained in the vivarium of VTCRI. To specifically obtain genetically labeled neurons, we crossed mice carrying Cre-recombinase under synapsin 1 promoter with an indicator line which expresses tdTomato upon Cre expression (B6.Cg-Gt(ROSA)26Sortm14(CAG-tdTomato)Hze/J).

### Lentiviral production

Mito-GFP sequence was sub-cloned into FUGW vector replacing the original GFP transgene. Lentivirus particles were produced in HEK293 cells using the third generation packaging system.

### Brain sectioning and immunofluorescent staining

Experimental samples were obtained from mice post-mortem. No experiments were performed on live animals for this study. Animals were deeply anesthetized to avoid any pain and sacrificed mechanically either by decapitation or exsanguination. The protocol was approved by Virginia Tech IACUC committee (IACUC #14–148). All animal procedures were performed in accordance with the guidelines for the animal care of laboratory animals issued by Virginia Tech. Three months old mature adult mice were deeply anaesthetized (ketamine/xylazine) and mouse hearts were canulated and perfused first with PBS (exsanguination) followed by 4% paraformaldehyde (PFA). The perfused mice were decapitated and brains were dissected out and fixed in 4% PFA overnight. The brains were cryopreserved by incubation in 30% sucrose solution for 48 hours. For sectioning, the brains were embedded in cryotek and 20 μm thick cortical sections were generated using a cryostat (IEC). The cortical sections were immunostained as floating sections; first they were permeabilized in 0.025% Triton X-100 and then blocked with 5% goat serum. Sections were stained with primary antibodies for two hours followed by secondary antibody incubation for 30 minutes. Finally, sections were mounted on slides using permafluor permount mounting medium (Thermo Scientific) and coverslips were sealed using nail polish.

### Neuronal culture

Primary cortical cultures were generated from P1.5 pups. The pups were decapitated and brains were dissected under aseptic conditions in Hanks balanced salt solution (HBSS). The cortices were isolated after careful separation of meninges. The isolated cortices were then minced and incubated with 0.25% trypsin at 37°C for 5 minutes. The cortices were washed with serum containing medium and triturated. Cells were spun down and resuspended in neuronal culture media (Lonza). Cells were cultured for 14 days *in vitro* (DIV) before immunostaining. Transfections in neuronal cultures were done using calcium phosphate method on 7 DIV. Neuronal cultures were also transduced with lentivirus on 7 DIV.

### Immunostaining of neurons

Neurons attached to poly-lysine coated coverslips were rinsed once with PBS and fixed for 15 min on ice in 4% PFA made in PBS. After fixation, the neurons were washed with PBS twice and then incubated for 30 min in blocking solution (i.e. PBS containing 5% goat serum and 0.025% Triton X-100) followed by 2 hour incubation with primary antibody and 15 min with Alexa 546- or Alexa 633-conjugated secondary antibodies (Thermo Scientific) respectively, diluted in blocking solution. The coverslips were then mounted on glass slides with permafluor permount mounting medium (Thermo Scientific) and analyzed using a Zeiss upright confocal microscope (LSM7) with a 63x/1.32–0.6 oil-immersion objective. Images were collected using Zen software and processed using Adobe Photoshop software. All digital manipulations were equally applied to the entire image.

### Electron microscopy

Cortical neurons cultured on coverslips were fixed for 30 min at 4°C in 2% glutaraldehyde buffered with 0.1 M sodium phosphate (pH 7.4). Coverslips were rinsed twice in buffer and incubated for 30 min at room temperature in 0.5% osmium tetroxide (OsO_4_). After rinsing with distilled water, coverslips were stained en bloc with 2% aqueous uranyl acetate for 15 min, dehydrated in ethanol, and embedded in poly/bed 812 epoxy resin (Polysciences) for 24 h. Sections (60 nm thick) were post-stained with uranyl acetate and lead citrate, and viewed with an FEI Tecnai transmission electron microscope at 120 kV of accelerating voltage. Digital images were acquired with an Olympus SIS Morada CCD camera.

### Serial block face scanning electron microscopy

Mice at P15 were anesthetized and transcardially perfused sequentially with PBS and 4% paraformaldehyde/ 2% glutaraldehyde in 0.1 M cacodylate buffer. Brains were immediately removed and hippocampi dissected. Tissues were then stained, embedded, sectioned and imaged by Renovo Neural Inc. (Cleveland, OH). Images were acquired from subiculum at a resolution of 5 nm/pixel and image sets included more than 200 serial sections (with each section representing 75 nm in the z-axis). SBFSEM data sets were 40 μm×40 μm×approximately 15 μm in dimensions. Two data sets were used for the analysis. Data sets were analyzed using TrakEM2 (http://fiji.sc/TrakEM2 website) [55]. Synaptic terminals were identified by the presence of synaptic vesicles and a close apposition of postsynaptic membrane.

### Brain subcellular fractionation

Brains from euthanized adult mice (euthanized by exposure to carbon dioxide followed by decapitation) were rapidly transferred to ice cold homogenization buffer (i.e. 0.32 M sucrose and 20 mM HEPES pH 7.4 with protease inhibitors). The brain was homogenized in a motorized homogenizer (~ 20 strokes). Whole brains were centrifuged at 1,000 g for 10 min to generate post nuclear supernatant (PNS). The PNS was centrifuged at 17,000 g for 15 min to obtain a pellet, which was subsequently washed once with homogenization buffer and then resuspended in homogenization buffer to obtain crude synaptosomes [40]. Crude synaptosomes were carefully layered over a percoll step gradient (ranging from 23% to 40%) and centrifuged to separate the mitochondrial, synaptosomal and myelin fractions as previously described [41].

### Mitochondrial respiration measurements

The mitochondrial oxygen consumption was measured in whole brain homogenates or crude synaptosomes using the conventional Clark electrode assay as previously described [56]. Briefly, the total respiration was measured in a buffer containing 0.3M mannitol, 10 mM KCl, 5 mM MgCl_2_, 10 mM KH_2_PO_4_ and 1 mg/ml BSA (pH7.4) in a water-jacketed cell magnetically stirred at 37°C (Hansatech instruments, Norfolk, UK). Oxygen consumption rates were measured both in the presence and absence of potassium cyanide (KCN) to assess the rate of KCN sensitive respiration.

### ATP measurements

The total ATP levels were measured using the kinaseglo kit (Promega). Briefly, the membrane extracts were freeze-thawed and mixed with kinaseglo reagent. The bio-luminescence was measured using a luminometer plate reader (BioTek). The ATP levels were normalized to the amount of total protein in each membrane compartment.

### Immunoblotting

Samples were separated by 10% SDS-PAGE, transferred to nitrocellulose, blocked in 5% skimmed milk for 2 hrs and incubated with primary antibody for 1hr followed by incubation with secondary antibody (1:5000 dilution) for 30 min at room temperature. The chemiluminescent signal was detected *via* the enzymatic reaction using ECL detection reagents (Amersham) and visualized on ChemiDoc (Biorad). For quantitative immunodetection, the blots were incubated with a fluorescent tag (alexa488) secondary antibody for 30 min at room temperature. The primary antibodies used included anti-ATP synthase subunit β (MitoSciences MS503, 1:1000); anti-synaptophysin (Sigma, 1:1000); anti-tubulin (DSHB, 1:1000) and anti neuN (Novus Biologicals, 1:1000).

### Protein quantitation

All protein quantitations were performed using the Coomassie Bradford reagent from Biorad, following the manufacturer’s instructions.

## Supporting Information

S1 Fig Cortical slices were incubated with mitotracker (green), postfixed and immunostained with synaptophysin antibody (red). Scale bar = 15μm.
(TIF)

S2 Fig Highly magnified image of brain sections stained with a mitochondrial marker Fis1 (red) and an active zone marker bassoon (green). Scale bar = 3μm.
(TIF)

S3 Fig Upper panel; Mito-GFP (green) was transfected in HEK cells, 48 hrs later cells were incubated with mitotracker (red) and imaged with Zeiss LSM. Scale bar = 5μm. Lower panel; Cells were transfected with dsRed (red) and mito-GFP (green), cells were counterstained with a mitochondrial marker ATP synthase β (blue).
(TIF)

S4 Fig Image of a neuron expressing mito-GFP, note the reticular network observed in the soma surrounding the nucleus. Scale bar = 5μm.
(TIF)

S5 Fig Example of low magnification images of HEK 293 cells and dense neuronal cultures indicating that almost all cells were transduced with mito-GFP lentivirus. Scale bar = 40μm.
(TIF)

S6 Fig Examples of low magnification images of low density neuronal cultures analyzed indicating that all neurons were transduced with mito-GFP lentivirus. Scale bar = 40μm.
(TIF)

S7 Fig High magnification of dendrites from low density neuronal cultures revealing large numbers of bassoon punctae (blue) do not colocalize with mitochondria (green) scale bar = 10μm. (TIF)

S8 Fig Lactate measurements from three independent preparations of whole brain homogenates and crude synaptosome. Data is plotted as mean and SEM.
(TIF)
