# Correction: Central Presynaptic Terminals Are Enriched in ATP but the Majority Lack Mitochondria

**DOI:** 10.1371/journal.pone.0181140

**Published:** 2017-07-10

**Authors:** Vrushali Chavan, Jeffery Willis, Sidney K. Walker, Helen R. Clark, Xinran Liu, Michael A. Fox, Sarika Srivastava, Konark Mukherjee

The Data Availability statement for this paper is incorrect. The correct statement is: Data are available from the figshare repository: (https://figshare.com/articles/data_for_uploading-1_pdf/5147311).

References are missing from the “Mice” subsection of the Materials and Methods section. The “Mice” subsection should read:

C57BL6 wild-type mice were maintained in the vivarium of VTCRI. To specifically obtain genetically labeled neurons, we crossed mice carrying Cre-recombinase under synapsin 1 promoter with an indicator line which expresses tdTomato upon Cre expression (B6.Cg-Gt(ROSA)26Sortm14(CAG-tdTomato)Hze/J) (Zhu et al., 2001; Madisen et al., 2010).

The references are:

Zhu Y, Romero MI, Ghosh P, Ye Z, Charnay P, Rushing EJ, Marth JD, Parada LF. Ablation of NF1 function in neurons induces abnormal development of cerebral cortex and reactive gliosis in the brain. Genes Dev. 2001;15:859–76. doi:10.1101/gad.862101.

Madisen L; Zwingman TA; Sunkin SM; Oh SW; Zariwala HA; Gu H; Ng LL; Palmiter RD; Hawrylycz MJ; Jones AR; Lein ES; Zeng H. 2010. A robust and high-throughput Cre reporting and characterization system for the whole mouse brain. Nat Neurosci 13(1):133–40. PubMed: 20023653MGI: J:155793.
