# Supplementary figures and images for: Central Presynaptic Terminals Are Enriched in ATP but the Majority Lack Mitochondria
Source: PLoS One. 2015 Apr 30;10(4):e0125185. doi: 10.1371/journal.pone.0125185 (PMC4416033; doi:10.1371/journal.pone.0125185)

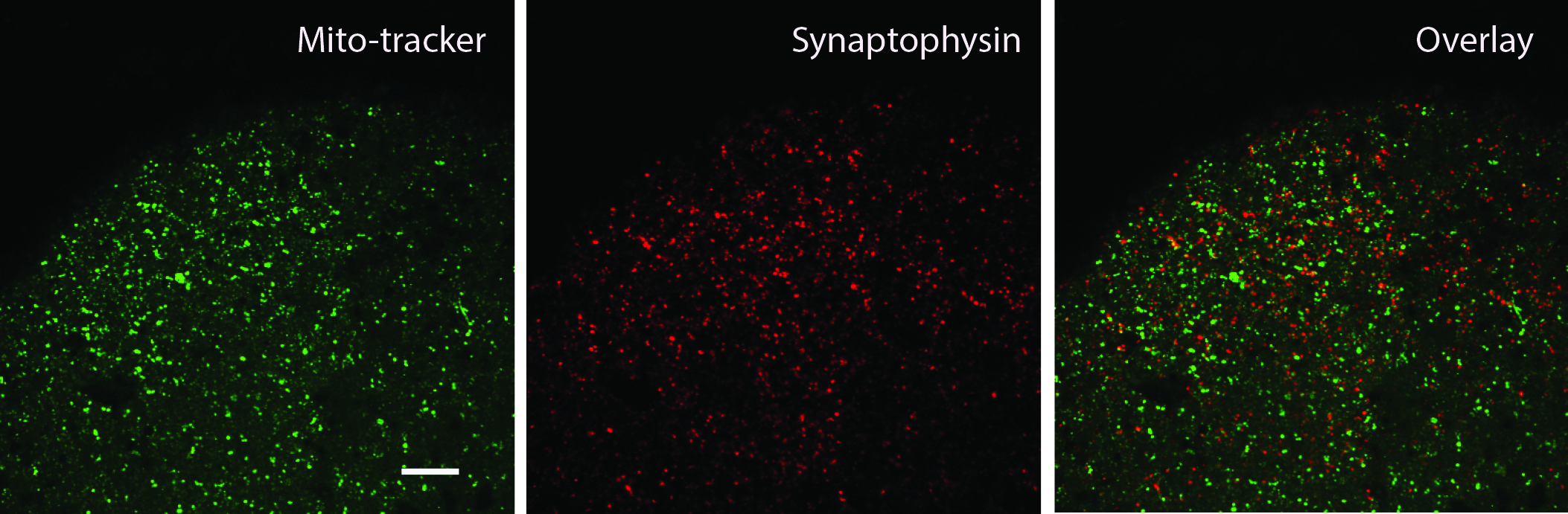

Supplement: S1 Fig — Scale bar = 15μm. (TIF) [file pone.0125185.s001.tif]

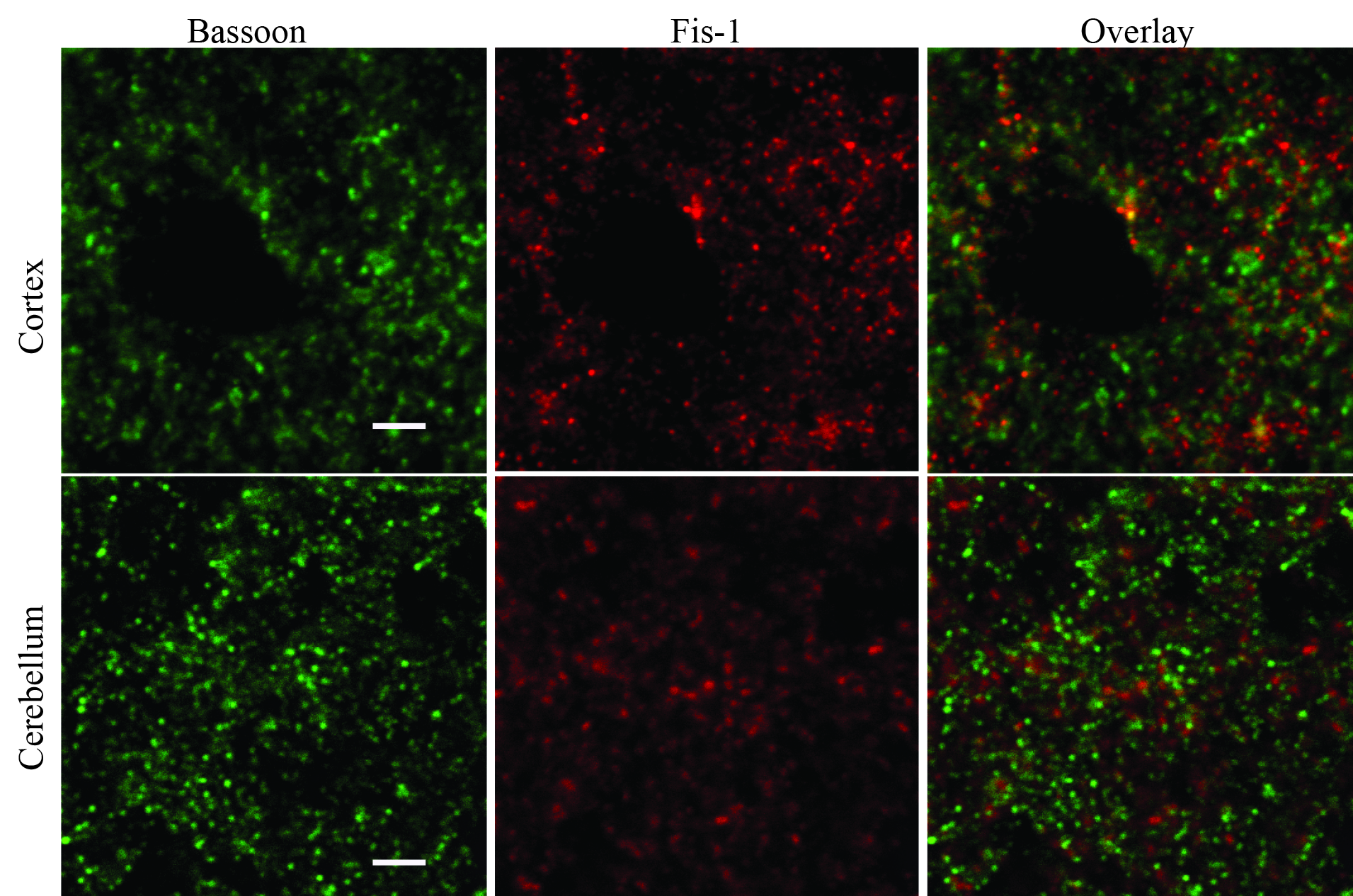

Supplement: S2 Fig — Scale bar = 3μm. (TIF) [file pone.0125185.s002.tif]

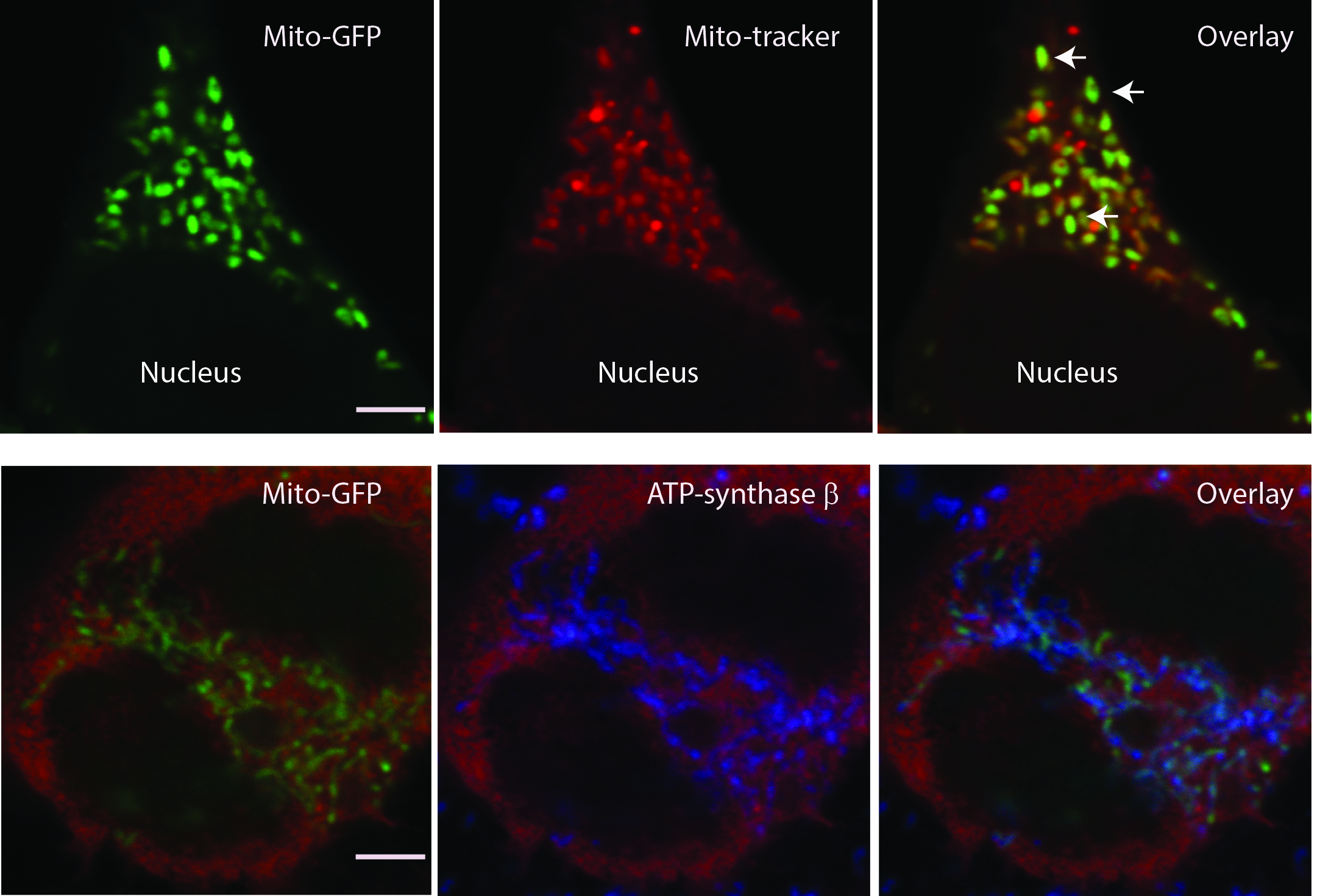

Supplement: S3 Fig — Scale bar = 5μm. Lower panel; Cells were transfected with dsRed (red) and mito-GFP (green), cells were counterstained with a mitochondrial marker ATP synthase β (blue). (TIF) [file pone.0125185.s003.tif]

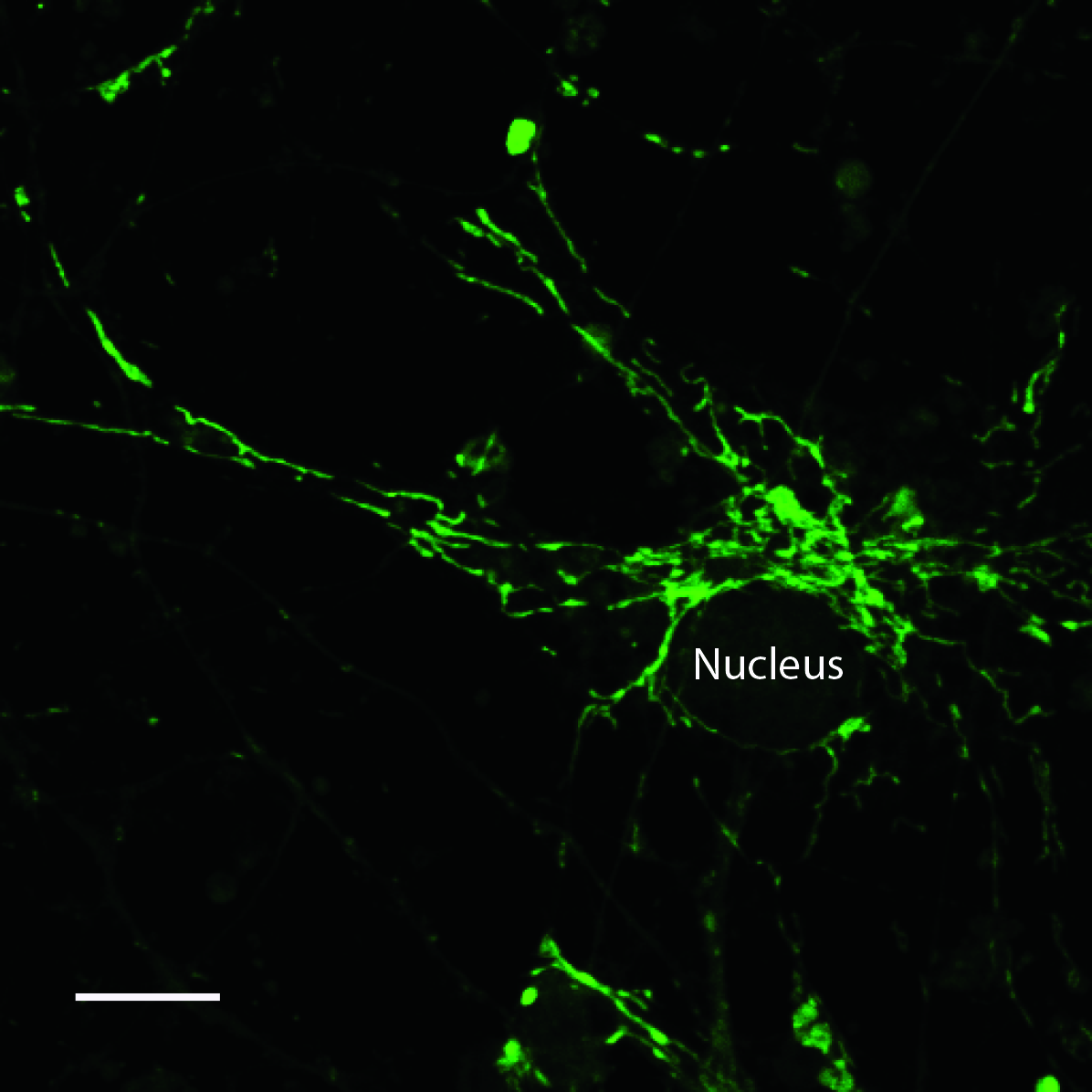

Supplement: S4 Fig — Scale bar = 5μm. (TIF) [file pone.0125185.s004.tif]

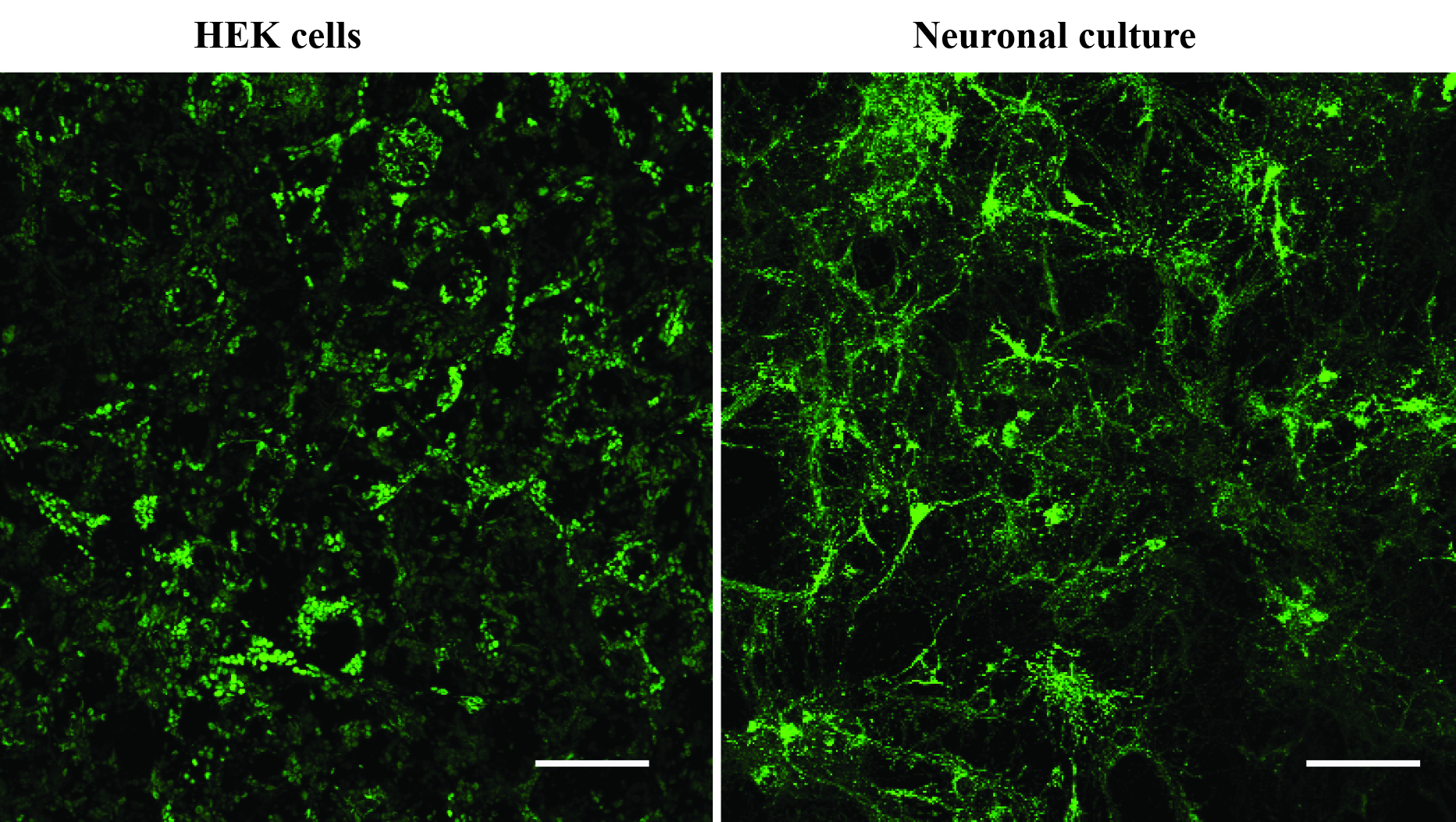

Supplement: S5 Fig — Scale bar = 40μm. (TIF) [file pone.0125185.s005.tif]

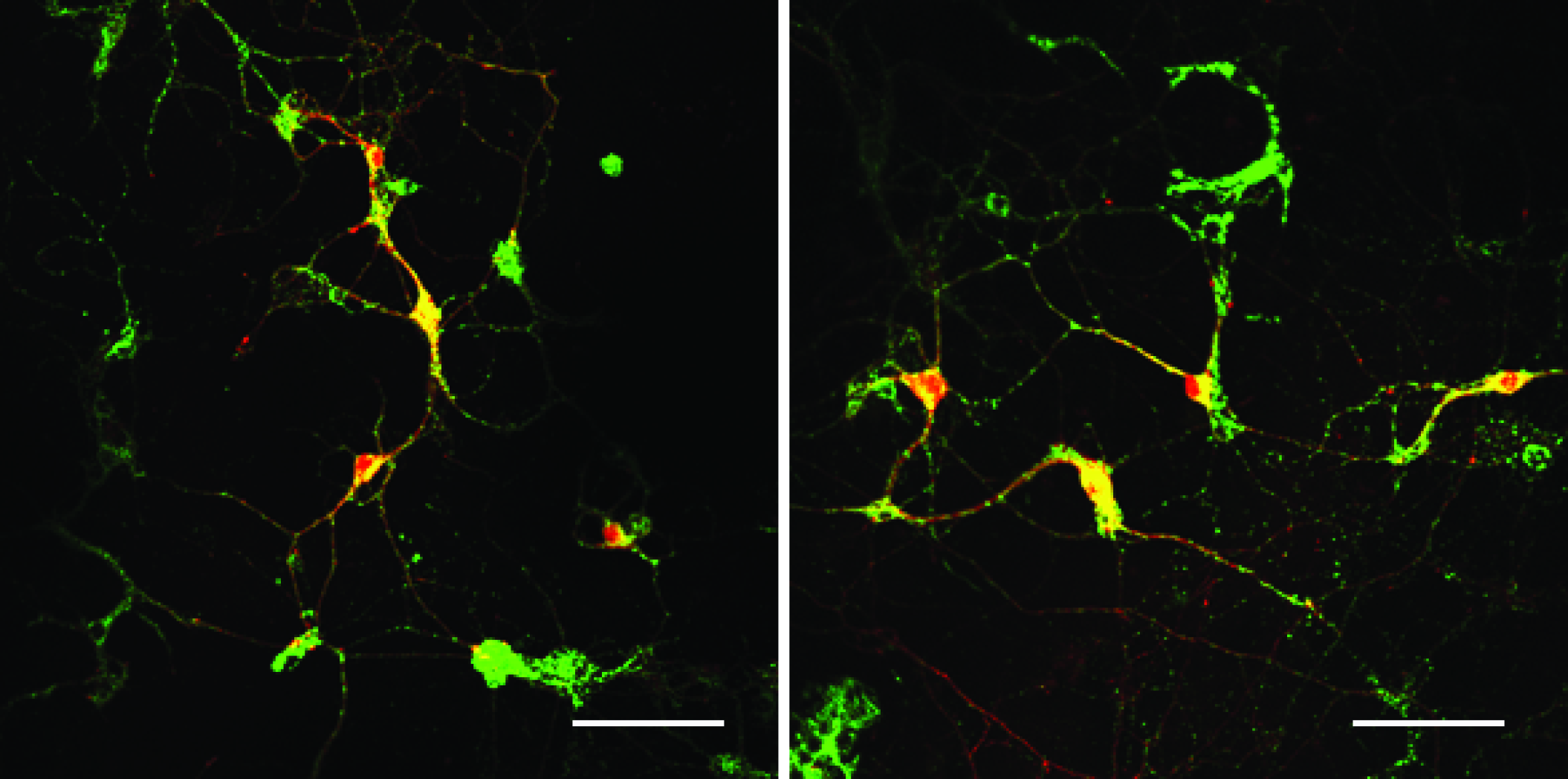

Supplement: S6 Fig — Scale bar = 40μm. (TIF) [file pone.0125185.s006.tif]

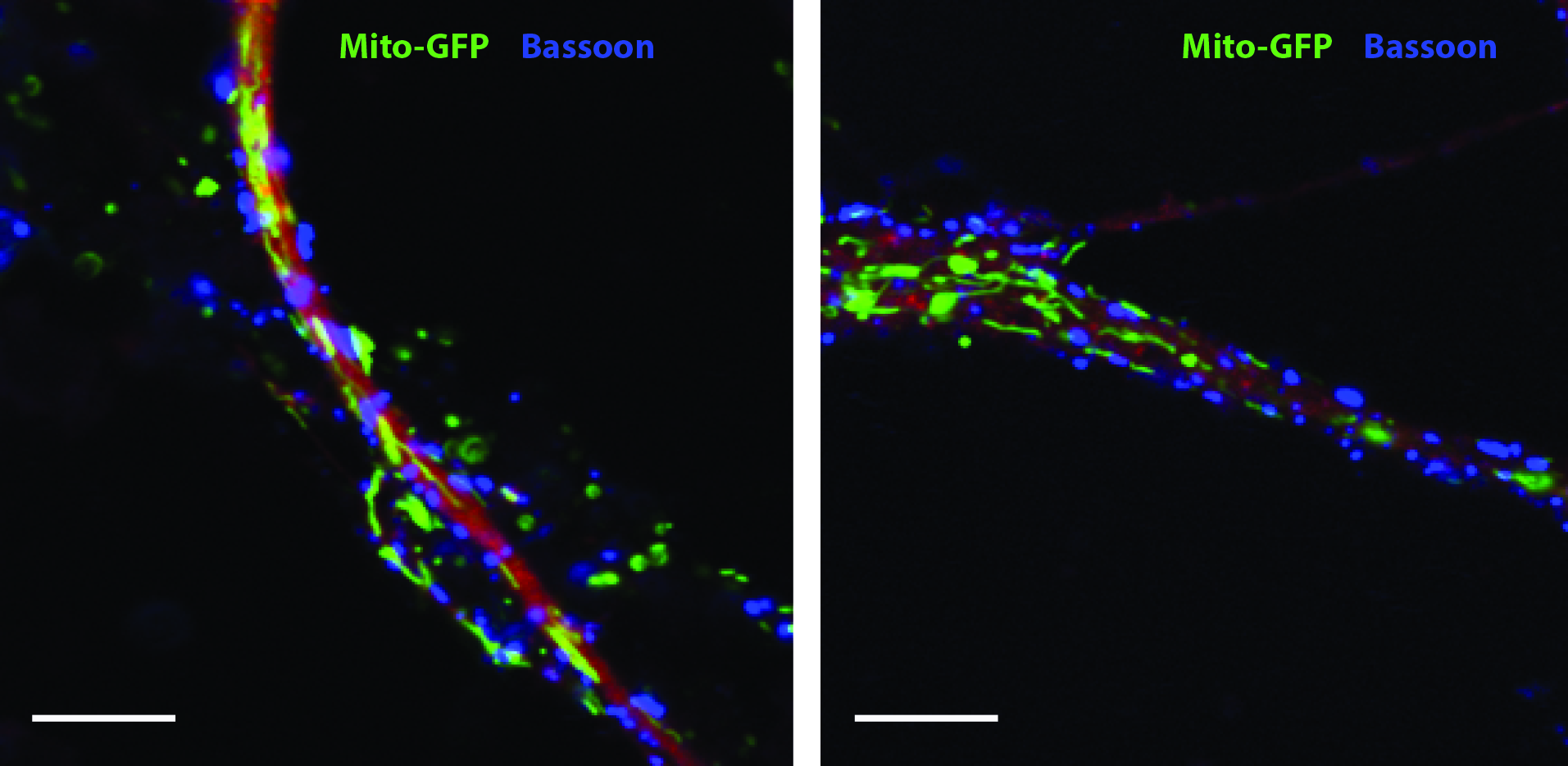

Supplement: S7 Fig — (TIF) [file pone.0125185.s007.tif]

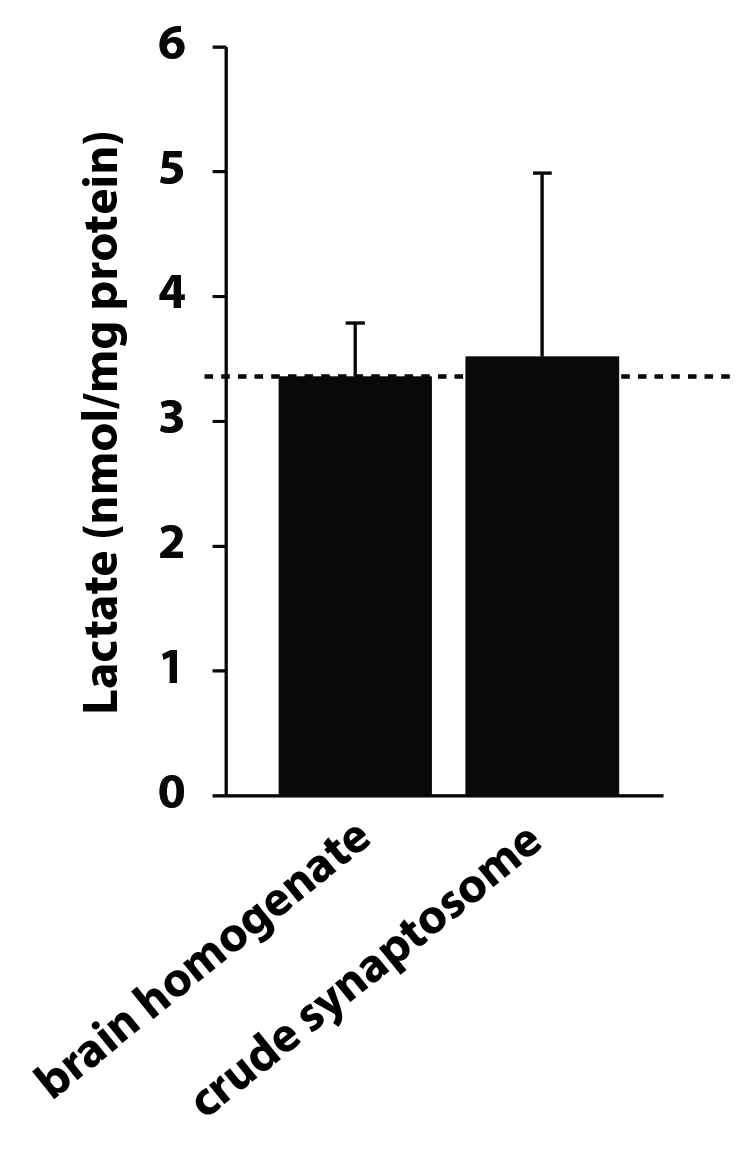

Supplement: S8 Fig — Data is plotted as mean and SEM. (TIF) [file pone.0125185.s008.tif]
